# Correction: Appealing perspectives of the structural, electronic, elastic and optical properties of LiRCl_3_ (R = Be and Mg) halide perovskites: a DFT study

**DOI:** 10.1039/d3ra90091f

**Published:** 2023-09-20

**Authors:** Nasir Rahman, Mudasser Husain, Vineet Tirth, Ali Algahtani, Hassan Alqahtani, Tawfiq Al-Mughanam, Abdulaziz H. Alghtani, Rajwali Khan, Mohammad Sohail, Abid Ali Khan, Ahmed Azzouz-Rached, Aurangzeb Khan

**Affiliations:** a Department of Physics, University of Lakki Marwat 28420 Lakki Marwat KPK Pakistan nasir@ulm.edu.pk; b State Key Laboratory of Mesoscopic Physics and Department of Physics, Peking University 100871 Beijing People's Republic of China 2201110247@stu.pku.edu.cn; c Mechanical Engineering Department, College of Engineering, King Khalid University Abha 61421 Asir Kingdom of Saudi Arabia; d Research Center for Advanced Materials Science (RCAMS), King Khalid University Guraiger Abha-61413 Asir Kingdom of Saudi Arabia; e Department of Mechanical Engineering, Taibah University Medina 42353 Kingdom of Saudi Arabia; f Department of Mechanical Engineering, College of Engineering, King Faisal University P. O. Box 380 Al-Ahsa 31982 Saudi Arabia; g Department of Mechanical Engineering, College of Engineering, Taif University P.O. Box 11099 Taif 21944 Kingdom of Saudi Arabia; h Department of Chemical Sciences, University of Lakki Marwat 28420 Lakki Marwat KPK Pakistan; i Magnetic Materials Laboratory, Faculty of Exact Sciences, Djillali Liabes University of Sidi Bel-Abbes Algeria; j Department of Physics, Abdul Wali Khan University Mardan 23200 KPK Pakistan

## Abstract

Correction for ‘Appealing perspectives of the structural, electronic, elastic and optical properties of LiRCl_3_ (R = Be and Mg) halide perovskites: a DFT study’ by Nasir Rahman *et al.*, *RSC Adv.*, 2023, **13**, 18934–18945, https://doi.org/10.1039/D3RA02640J.

The authors regret that the name of one of the authors (Vineet Tirth) was shown incorrectly in the original article. The corrected author list is as shown above.

The Royal Society of Chemistry apologises for these errors and any consequent inconvenience to authors and readers.

## Supplementary Material

